# Neck dissection in squamous cell carcinoma of the larynx. Indication of elective contralateral neck dissection

**DOI:** 10.1590/S1808-86942012000200002

**Published:** 2015-10-20

**Authors:** Ali Amar, Helma Maria Chedid, Sergio Altino Franzi, Abrão Rapoport

**Affiliations:** a PhD – UNIFESP (Head and Neck Surgeon at the Heliópolis Hospital); b MSc – HOSPHEL (Head and Neck Surgeon at the Heliópolis Hospital); c PhD – USP (Head and Neck Surgeon at the Heliópolis Hospital); d Senior Associate Professor – USP (Head and Neck Surgeon at the Heliópolis Hospital)

**Keywords:** carcinoma, squamous cell, larynx, neck dissection

## Abstract

Unilateral or bilateral neck dissection must be considered in the treatment of laryngeal cancer

**Aim:**

To evaluate the prevalence of contralateral metastases in larynx cancer and distribution of these metastases according to lymph node levels in the neck.

**Method:**

Retrospective longitudinal study of 272 charts from patients with squamous cell cancer of the larynx treated between 1996 and 2004; and we selected 104 surgical cases submitted to neck dissection. We evaluated the incidence of bilateral or contralateral metastases, according to the location and extension of the primary tumor, considering the anatomical sub-sites and the midline.

**Results:**

Contralateral metastases in lateral tumors were observed in 3.5% of glottic lesions and in 26% of supraglottic lesions. Contralateral metastases were uncommon in N0 patients. Lymph nodes levels IIa and III were the most commonly involved in the neck.

**Conclusion:**

In lateral glottic tumors there is no need for elective contralateral neck dissection. In supraglottic lesions without ipsilateral metastases, the incidence of hidden metastasis does not justify elective contralateral dissection. The midline is not a reliable indicator of the risk of contralateral laryngeal tumors.

## INTRODUCTION

Lymph node metastases are the main prognostic factor in the upper air and digestive tracts, and elective neck dissection – more than a staging procedure, enables high rates of regional disease control^1^. Although lymphatic drainage and the risk of metastasis in each anatomical site have been established, the indication for elective contralateral neck dissection still is controversial^2^. The extension of this dissection has impacts both on treatment morbidity, mortality, and in disease control. The goal of the present study is to assess the prevalence and location of lymph node metastases in laryngeal tumors, according to primary tumor extension, considering the indication of contralateral neck dissection.

## MATERIALS AND METHODS

We reviewed the chart from 272 patients with laryngeal epidermoid carcinoma, consecutively seen between January of 1996 and December of 2004, including 104 patients submitted to surgical treatment with neck dissection. We did 161 neck dissections – 65 were complete and 96 were selective. Primary tumor extension was determined based on the intraoperative descriptions, considering the anatomical sub-sites involved and their relationship with the median line. Thus, the lesions were classified into two groups: lesions with their center in the median line and lateralized lesions, the latter were further divided according to their relationship with the median line. We considered the lymph node levels involved in the pathology exam, according to the classification from the American Head and Neck Society, as well as the isolated regional recurrences which may eventually occur. The lymph node level classification was done by a surgeon of the team, immediately after the procedure, and they were sent separately for histological analysis. All the patients were re-staged according to the TNM-2002 classification from the UICC-AJCC, considering the physical exam upon admission. The statistical analysis used descriptive methods and the qualitative analysis used the Fisher's Exact Test, considering significant those differences with an alpha error lower than 5%.

## RESULTS

The mean age was 56 years (36 to 79) – 86 males and 18 females. As to the anatomical subsite: 66 lesions were glottic, of which 40 were transglottic, 35 supraglottic and three were extensive lesions with unidentified site of origin. As far as staging is concerned, three patients were in stage I; 19 in stage II; 45 in stage III and 37 in stage IV. The mean follow up of asymptomatic patients was 58 months, and only six cases had less than one year of follow up.

Among the 104 cases, 53 (50,96%) had histologically confirmed metastases. Bilateral metastases happened in 22 (21%) cases. Primary tumors with epicenter in the median line or extensive tumors involving the entire organ, added to 28 cases; 15 (53%) with bilateral metastases. In the 76 cases of lateralized tumors, there were bilateral metastases in seven (9%) – and hidden in four cases. There was no case of isolated contralateral metastasis.

In the 76 cases of lateralized lesions, only 29 were submitted to bilateral dissection. Of the 47 remaining patients, 12 underwent postoperative radiotherapy, including the contralateral neck. There were two (3%) cases of bilateral metastases in 59 NO patients; and five (29%) cases among the 17 N+ patients (*p*=0.005).

Among the 57 patients with lateralized glottic lesions, there were two cases (3%) of bilateral metastases, both clinically identified. Among the 19 patients with lateralized lesions in the supraglottis, we observed bilateral metastases in five (26%), *p*=0.009; and only one was found upon physical exam. The epiglottis was involved in 15 of these patients, and all five patients with bilateral metastases had epiglottis involvement. There were nine neck recurrences, with only one case in which the contralateral neck was not dissected.

The T staging did not significantly influence the presence of bilateral metastases in the lateralized tumors. We found bilateral metastases in 1/19 (5%) of the T1 and T2 cases and in 6/57 (10%) of the T3 and T4 cases; *p*=0.67.

The metastases were predominantly in levels II and III (Table 1). Level IV metastases were found in three cases; however, level IV was not routinely separated from the surgical specimen and is underappreciated in this sample.

**Table 1:** Lymph node levels involved according to the type of neck dissection (n=104).

Neck Dissection			
Level	Elective*	Therapeutic#	Total
I	1	2	3
IIa	29	29	58
IIb	1	2	3
III	11	21	32
IV	3	3	6
V	0	4	4

* 117 dissections in one side of the neck – N0.

# 44 dissections in one side of the neck – N+.

## DISCUSSION

The low morbidity of the selective dissection enabled us to broaden the indication for elective dissection, although the bilateral neck dissection may multiply the risk of complications with the exposure of both neck vessel-nervous bundles. In this series, approximately 50% of the patients were submitted to a contralateral elective dissection. Neck dissection and post-operative radiotherapy yielded regional disease control in most of the cases, which prompts for a discussion about overtreatment possibilities. Glottic lesions, including the transglottic, have a low prevalence of contralateral metastases, even if the primary tumor goes beyond the mid line. The results of the present study are similar to the ones reported by Marks et al.^2^. In supraglottis tumors, involvement of the central region established a high risk of contralateral metastases. In these cases, the incidence of contralateral metastases in lateralized lesions is higher than 20%, but are infrequent in the absence of ipsilateral metastases^3^, ^4^. The ipsilateral pathological staging (pN) is even more reliable vis-à-vis the risk of contralateral metastases. Almost half of the patients with supraglottic tumors had central tumors, such a situation that requires bilateral treatment. In lateralized lesions, when there are histologically confirmed metastases in the ipsilateral side, contralateral metastases happen in up to 48% of the cases, while in stages I and II this frequency is of only 5%^5^, ^6^.

Although the T staging (TNM – UICC/AJCC) is very important in the definition of the risk of ipsilateral metastases, its relationship with the risk of contralateral metastases seems to be indirect, considering that the tumor size is associated to the possibility of reaching areas (anatomical subsites) with crossed lymphatic drainage. In the present study, the T stage alone did not prove applicable in indicating contralateral elective dissection – a result which was also observed by other authors in supraglottic tumors^7^, ^8^.

The possibility of postoperative radiotherapy, especially when the characteristics of the primary tumor and ipsilateral metastases would justify the adjuvant treatment, also deserves to be considered in relation to its use in the treatment of the contralateral neck. Although the efficacy of radiotherapy in the treatment of subclinical regional disease is an indirect evidence, the results are in favor of its use in this context^1^, ^4^, ^9^. Based on principles, the initial treatment must be sized in order to completely treat the disease, while the adjuvant treatment may be used for areas of a greater risk of recurrence, acknowledging that the advanced disease requires a combined treatment. Elective dissection is usually indicated when the risk of hidden metastases is higher than 20%; nonetheless, since in laryngeal surgery both jugular and carotid bundles are exposed, a dissection of opportunity may be indicated in situations in which the risk of metastasis is lower^10^. By the same token: comorbidities, the ease of patient follow up and the speed of rescue of a regional recurrence must also influence the decision to surgically treat the contralateral side. Some recurrences already present themselves as non-resectable disease and elective dissection provides the best regional control^4^, ^6^, ^11^, ^12^, ^13^. The results from the present study have shown that approximately 40% of the elective contralateral dissections, especially in NO supraglottic or glottic tumors, could be avoided without impairing the staging, since they proved to be pN0, but it is not possible to establish if the omission in neck dissection could affect the excellent result obtained in the regional disease control or even patient survival^4^, ^13^. We cannot disregard that in the routine pathology assessment there may be 8% false pN0, since one single cut in the lymph node fails to detect micrometastasis^14^.

As to the extension of the dissection, the metastases are concentrated in levels IIa and III, which represent the first drainage stage of these tumors, enabling a super-selective dissection only of these levels in a negative neck^8^, ^15^, ^16^, ^17^. Isolated metastases outside of this level are rare, and happen in only around 1% of the cases.

## CONCLUSION

We concluded that there is a greater likelihood of bilateral metastasis in extensive tumors with involvement of the entire organ or with its epicenter in the median line. Glottic tumors have low frequency of metastases, regardless of crossing the mid line. In cases of lateralized tumors, the presence of N+ neck and the involvement of the epiglottis were significant in the presence of bilateral metastases.
